# Commentary: The trend towards devolution in clinical chemistry

**DOI:** 10.1155/S1463924679000511

**Published:** 1979


### Commentary

### The trend towards devolution in clinical chemistry

Amongst the many branches of chemistry, that associated with health care is unique for several reasons. It is more personal because it has to be done for the individual alone. Unlike the industrial chemists in food factories or sewage works, the clinical chemist works for single members of a population. Therefore, he has more scope for his efforts in numerical terms throughout the world than his colleagues in other branches. Indeed, if full chemical analytical coverage to currently accepted standards were made available worldwide, four billion persons would require an army of some 800,000 scientists together with laboratories and equipment.

The numbers game and the fact that assays are largely carried out on one type of material in clinical chemistry – blood and its constituents which are often in extremely low concentrations – have led to many analytical firsts for medical scientists. These include laboratory automation, gas chromatography and radioimmunoassay to mention only a few. The post-war explosion in work-loads necessitated the introduction of automation. The cost and size of the machines which were rapidly developed as a result brought centralisation of analytical department. The large central laboratory flourished because it provided advantages for the types of quality control schemes which have been developed over the last fifteen years. Both computerised and non-computerised data handling and storage systems were more easily developed for central laboratories and large centralised workloads allowed the appointment of specialised staff to interpret data.

Centralisation, however, has its problems. Unless a specimen is to be classified as an emergency, it has to enter the laboratory organisation machine where specimen transport, data logging, analysis, reporting and report transport are costly in time and money. There is no doubt that if some of the assays could be carried out near the patient, simply, rapidly and accurately, the advantages to both the patient and the clinician would be considerable. On-the-spot decisions could be made on wards. In the outpatient departments patients might be diagnosed and treatment commenced without the awaiting of results requiring further visits. The GP would also find many advantages if he were able to do chemical analyses in his surgery or while on domicillary visits.

Scrutiny of the work-loads of the large centralised laboratories shows that only about ten different types of assay account for 85% of the total performed. This small qualitative but large quantitative part of the work contributes much to the present tendency towards considering large laboratories more as factories than as scientific departments. There is some truth in the claim by technical staff in various countries that with their increasing management experience they can run such centres as well as, or better than, those who profess to be scientists. Centralisation took place because the trend of technological development led to the highest efficiency being produced using large instruments with large workloads. It is now apparent that accuracy, precision and cost effectiveness are not the sole prerogative of the large machines and centres. Their advantages are slowly diminishing for several reasons.

(1) The cost, size and efficiency of solid-state electronics and data processors allows them to be used in small machines with dramatic effect.

(2) The use of pre-packaged reagents has shown the value of the high level quality control which is possible in the commercial mass production of this material.

(3) Reliance upon a single large machine which is inevitably liable to breakdown has caused problems on many occasions.

(4) Large machines in the past have been criticised for their inflexibility.

(5) Time and effort can be expended in organising the conveyance of specimens to the central laboratory, and the distribution reports therefrom.

(6) Biochemical profiling, where many assays are carried out on an individual, regardless of whether or not they are indicated by illness, require the development of large machines, but the extent of the value of such profiling has recently been widely questioned.

(7) Funds cannot be readily acquired to cover the cost of equipping and running large centres for much of the world's population, nor are the maintenance arrangements and suitable electricity supplies available.

(8) Exciting new techniques for example layer chemistry, hold considerable potential for application to small instruments operated by relatively inexperienced individuals working in relative isolation.

The new generation of instruments suitable for devolution can cover a variety of situation. One answer for the small independent laboratory has been available for the last decade. This is the principal of high quality pre-packaged chemistry, as used in the Du Pont ACA, or more recently in the Technicon STAC. Realisation of the potential that this approach could have has proceeded extremely slowly. This undoubtedly accounts for the slow but steadily increasing sales of an instrument, conceived 10 years ahead of its time. When such a machine is installed in a ward environment or similar situation near to the patients it is meant to serve, the high costs of its reagents are counter-balanced by the somewhat imponderable savings produced by speedy results increasing the efficiency of the clinician and the convenience of his patients, and eliminating the overhead costs of the large laboratory.

It could be argued that this method of devolution will make it difficult or impossible to operate an efficient data processing or data banking system, the value of which has become apparent in the overall operation of the centralised laboratory. Modern computer systems with distant terminals now make it possible for input and output devices, connected to a central processor, to operate at almost any distance. Even if on-line operation is not appropriate, off-line cassette tape recording can be an effective system of communication. If a direct link to a central processor is feasible, the processing power available to a small distant instrument is almost unlimited and in this way many of the restrictions enforced on the chemistry operable on such a small instrument can be overcome.

Several automatic colorimeters are now available for use where the sophistication and cost of fully automated packaged chemistry instruments are not appropriate. They may be pre-programmed or programmable for a large range of assays; their use removes a fair proportion of the chances of human error which are associated with optical density reading and the subsequent calculations. For isolated and developing country situations, this approach is at present probably the most ideal, but instruments must be simple to operate, capable of maintenance-free running for many years and have a power consumption such that they can function for long periods on an integral battery.

For satisfactory operation, all such automatic colorimeters must be used in association with equally simple-to-use trouble-free chemistry. The development of new technologies, together with a need to serve a larger proportion of the world's population, must of necessity produce changes, and rational argument would appear to point away from centralisation; just as sure as the automation of clothes washing first led to big central laundries with large capacity machines, and then to their partial demise following the evolution of the highly efficient home washing machine and community launderette.

*F.L. Mitchell*

### From the Editor's desk

This issue marks a significant point in the Journal of Automatic Chemistry's life, the final issue of the first fornative year, but Volume 1 will be completed with the October issue. Whilst there has been a healthy supply of papers being submitted for consideration in the Journal there has not been a flood of letters from the readership. Those letters we have received express the value of the new journal and they encourage us to continue along the lines established throughout the first year. In general the breakdown of readership is approximately divided equally between those with clinical and those with industrial interests. My own colleagues with an industrial background find considerable use reading all the articles so it is worthwhile not being put off by the title of the paper or by the affiliation of the authors. There is considerable similarity in approach and problem between work within an organisation such as the Clinical Research Centre and work relating to the analysis of tobacco smoke at the Laboratory of the Goverment Chemist. There is a great deal to be gained by communications across disciplines – it is too easy to say that there is far too much to read on one's own subject area. However, there is a common interest in instrumentation and it is vital that the advantages of microprocessors and new technical developments are integrated correctly into our working lives. This I submit, requires a collective objective.

In his editorial commentary Dr. Mitchell discusses the shift from centralised power to local control. This is a very important change, however it does not detract from the value that can be gained by a centralised facility, but it does more easily put the control where it ought to be. Any user of a computer system wants to feel that he is in complete control. How this is achieved is not important but many of the centralised approaches in the past have failed to meet this objective. Successful manufacturers are producing instruments more in line with the market needs. The control of the instrument must be situated with the instrument. However, it is vitally important that such instruments are able to communicate with each other and with a central intelligence. The first attempts by Hewlett Packard to introduce microprocessor technology into their automated gas chromatographs did not achieve a great degree of acceptance, they were unable to talk simply to other computers. The second generation from the same company has not a similar fault, is expandable and represents a significant advance in the first attempt. With such advantages there are really two problems that must be overcome. It is important to standardise interfaces so that it is a relatively simple matter of a connection to other instruments or computers. There is also a need to generate truly portable software packages. Software is a subject which is generally glossed over in the literature but it is a very costly item which must be integrated into the cost benefit equation. Many instrument manufacturers absolve themselves from this discussion by offering a basic computer on the grounds that everybody will be able to 'speak' basic in the near future.

The paper by Professor Bonner Denton published in the third issue of the Journal of Automatic Chemistry attempts to overcome one part of the problem, that of control. The availability of cheap development computers such as the Commodore PET is also seen by some as a way out of the problem. However, in my own experience, whilst the hardware is generally a quantifiable aspect of the problem, the software in terms of specification and production is both more expensive and difficult to obtain in acceptable timescales.

At the 3rd European Congress of Clinical Chemistry at Brighton, (a report of this appears on page 226), I was able to hold an editorial meeting with my clinical corresponding editors who had assembled for the meeting. This was of considerable value and many useful suggestions helpful to the progress of the Journal emerged. One important point was that the meeting complemented the face to face meeting I have had with each member of the editorial team. This will make it much easier in future to correspond between ourselves and will I am sure increase the flow of information. Perhaps the most useful point that emerged is that Dr. Collombel suggested that a glossary of published evaluation reports should be published in the Journal on a continuing basis. A retrospective list of all instrument evaluations currently available will be published and periodically will be updated. In conjunction with the references a short abstract of the evaluation will be provided.

Clinical chemists place considerable value on evaluation reports, and have established a protocol for such evaluations. In industrial applications such reports are not in vogue. They are often confined to intra-organisational reports and not published in the scientific literature. However, with considerable pressure on available resources it is hoped that some standardisation of evaluation report in the industrial market can emerge. Certainly it seems that this is one area where industrial chemists can learn from our clinical colleagues. One useful role of the Journal is to publish evaluation reports and to do this in a quick timescale. It is an important factor in the communication of information from manufacturers to users and vice versa.

*Peter B. Stockwell*

